# Understanding the mechanisms of harm: Crisis pregnancy centers and reproductive health in the post‐Dobbs era

**DOI:** 10.1002/pmf2.70479

**Published:** 2026-10-04

**Authors:** Ashish Premkumar, Hillary McLaren

**Affiliations:** ^1^ Department of Obstetrics and Gynecology Biological Sciences Division University of Chicago Chicago Illinois USA

For decades, crisis pregnancy centers (CPCs) have been part of the American landscape of reproductive health [1]. A 2013 analysis by Hussey demonstrates that up to 4000 CPCs are operational within the United States, outstripping the number of freestanding abortion clinics by at least a factor of 3 [2]. CPCs make a multitude of claims regarding their public‐facing role—ranging from providing needed supplies for newborn care to offering counseling about reproductive options with an explicit antiabortion lens [3, 4]. Data derived from interviews with CPC leaders serving communities with limited obstetrical services demonstrate a generalized lack of implementation of evidence‐based health and social risk screening strategies, which can exacerbate inequities in reproductive health outcomes among marginalized communities [5]. While CPCs are not categorized as medical care providers, they play an outsized role in limiting abortion access and engaging in forms of coercive counseling about reproductive choice, which we broadly categorize as a form of reproductive violence [6].

Data demonstrate that CPCs delay an individual's engagement with abortion care and, if desired, entry into prenatal care [7, 8]. Though these upstream factors may be broadly defined in the literature, they have substantial impacts on the health and well‐being of reproductively capable people. First, delays in access to abortion care may limit access to local abortion services, thereby requiring out‐of‐state travel with significant financial, social, and psychological hardships [9, 10, 11, 12]. Further, delays in abortion care have been associated with increased morbidity at the time of abortion, primarily driven by gestational duration at the time of abortion [13]. Second, delays in initiation of prenatal care, especially among individuals who may have comorbidities which place them at high risk of maternal morbidity or mortality, can have substantial impacts on maternal health [14]. However, at the heart of both issues is the derailment of reproductive autonomy. Carrying an undesired pregnancy has substantial health ramifications for the pregnancy‐capable individual and, when compared to abortion care, increases the risk of morbidity and mortality [15, 16].

Despite these issues with CPCs, data are limited in understanding how CPCs engage in deceptive discourse in the public space [17]. In the manuscript by Stimpson et al., the research team conducted a mixed‐methods analysis of Instagram posts related to CPCs. The authors identified four overarching themes within the content: Service Promotion & Outreach, Emotional Appeals, Ideological Messaging, and Misinformation & Misrepresentation. Although posts categorized as Misinformation & Misrepresentation comprised the smallest proportion of the sample (10%), they generated the highest levels of engagement (as measured by likes and comments). Regardless of motivation behind posts, misleading content appears to be most engaging to the public, potentially contributing to misconceptions about CPCs and their role in the reproductive landscape.

These data shed light on the sociological mechanisms by which CPCs can lead to reproductive harms on a public forum. These data are critical for clinicians, advocates, legislators, and researchers alike to contextualize the multifaceted ways public health messaging on maternal morbidity and mortality can be undermined to the detriment of reproductively capable individuals across the United States. As reproductive autonomy is being eroded at the state and federal level, we call upon researchers to use novel, multidisciplinary methodologies to investigate the impact CPCs and other coercive forms of abortion restrictions (e.g., institutional restrictions on abortion at faith‐based medical organizations [18]) have on maternal morbidity and mortality.

## AUTHOR CONTRIBUTIONS

**Ashish Premkumar**: Conceptualization; writing—original draft. **Hillary Mclaren**: Conceptualization; writing—original draft.

## CONFLICT OF INTEREST STATEMENT

The authors declare no conflicts of interest.

## Data Availability

Data sharing is not applicable to this article as no datasets were generated or analyzed during the current study.
